# Primary meningococcal arthritis in a COVID-19 18-year-old man: a case report and review of the literature

**DOI:** 10.1186/s12879-021-06211-7

**Published:** 2021-05-29

**Authors:** Norman Ducatez, Marine Chancel, Youcef Douadi, Charles Dayen, Rémi Suguenot, Emmanuelle Lecuyer, Benoit Brihaye, Houcine Bentayeb

**Affiliations:** 1 Pneumology and Infectious Diseases department, Saint-Quentin hospital, 1 avenue Michel de l’hospital, 02100 Saint-Quentin, France; 2 Internal Medicine department, Saint-Quentin hospital, 1 avenue Michel de l’hospital, 02100 Saint-Quentin, France

**Keywords:** Arthritis, COVID-19, *Neisseria meningitidis*

## Abstract

**Background:**

SARS-CoV-2 (severe acute respiratory syndrome coronavirus 2) infection is associated with various complications. PMA (primary meningococcal arthritis) is a rare meningococcus-associated disease causing arthritis of the knee usually, without any signs of invasive meningococcal disease. No case of PMA in a COVID-19 (coronavirus disease, 2019) patient has yet been described. PMA mainly strikes young adults. PMA is not associated with any immunocompromising condition. It has a better outcome than usual septic arthritis

**Case presentation:**

Herein, we report an 18-year-old man diagnosed with COVID-19, later admitted with persistent fever, right knee arthralgia and maculopapular rash. Due to family history, psoriasis and Henoch-Schönlein purpura were hypothesized and ruled out. Finally, synovial fluid culture confirmed *Neisseria meningitidis * serogroup B arthritis without any other symptoms of invasive meningococcal disease. Healing was achieved quickly with surgery and antibiotics. We concluded in a PMA*.*

**Conclusion:**

We describe here the first primary meningococcal arthritis in a COVID-19 patient and we hope to shine a light on this rare but serious complication.

## Background

Worldwide, 10% of human beings are considered asymptomatic carriers of *Neisseria meningitidis* [1]. It can be a potentially aggressive bacteria, causing invasive and possibly deadly infections such as meningitis or septicemia [1, 2]. Indeed it is known to cause fever, headache, nausea, vomiting, severe myalgias, nonspecific rash, sore throat and upper respiratory symptoms. Alongside the well-known meningitis and purpura fulminans, some presentations are far rarer, such as primary meningococcal arthritis (PMA) and chronic meningococcemia [3]. Bloodstream dissemination mechanism is unknown.

In literature, PMA has three peaks of incidence, the early years of life, around the age of 20 and over the age of 65, with the highest prevalence for the young adult peak [3]. The most common presentation is a monoarthritis of the knee, which is, by far, the most involved joint. The most found serogroup is *N.meningitidis * B [4]. No underlying condition, in particular no immunocompromising condition has been proved to facilitate PMA [3]. Lavoipierre et al. reported a case of a young woman with no medical history but with a family history of inflammatory rheumatism [5] that could indicate an underlying genetic susceptibility. Nonetheless some similarities can be found in the reported cases, such as age, medical history, and clinical presentation. Young people, male or female [6] with no medical history are most represented [2–4], but we can also find several cases over the age of 60 [3, 7]. The knee is very often involved, either as monoarthritis or as part of an oligoarthritis. The prognosis is generally good [3] unlike arthritis usually caused by Gram positive Cocci, (i.e. *S.aureus * or *Streptococcus spp*), or less often by Gram negative Bacilli. These infections are accompanied with morbidity, limited functional use of the impaired joints and reduced quality of life. Nowadays, it is acknowledged that a quick treatment, including surgical joint drainage and initial intravenous antibiotherapy reduces such consequences [2]. Up to 2019, only 47 cases of PMA have been reported in PubMed [3]. Finally, cases of acute arthritis during COVID-19 were reported [8–10] but none involving *N.meningitidis.*

Here, we report the case of a primary meningococcal arthritis without any other typical symptoms of meningococcemia in a young COVID-19 male patient.

## Case presentation

The patient is an 18-year-old man with no medical history, no usual medication nor drug abuse. Familial history revealed a possible psoriasis in the mother, a possible Crohn’s disease in the uncle and Henoch-Schönlein purpura in the sister. There was no relevant traveling exposure, nor any evidence of zoonotic contamination such as tick bites. There was no unprotected sexual exposure, nor any contact with ill people, within the prior weeks. Lastly, no trauma was reported.

On day 1, the patient consulted his family doctor for fever, asthenia, myalgia, headache, erythema of hands and feet and burning sensation during urination. On day 2, the retro-transcriptase polymerase chain reaction (RT-PCR) for SARS-CoV-2 was positive, and the patient stayed home awaiting improvement. On day 8, he presented with a painful and swollen right knee, and a worsening asthenia and fever, but an improvement in respiratory and cutaneous symptoms. On day 14, he consulted a rheumatologist at our hospital in the city of Saint-Quentin, France, for persistent pain in the right knee, but physical examination was strictly normal.

On day 15, the patient was admitted in our Internal Medicine department as signs of arthritis were found upon new examination of his right knee. The other joints were normal, the backbone was painless. Respiratory examination showed full recovery of COVID-19. Cutaneous examination showed *livedo reticularis* on the feet, petechiae on the bottom-half part of legs and arms. There were no signs of psoriasis. Abdominal examination was normal, no diarrhea was reported. Neurological examination was normal, no meningeal syndrome was found, therefore lumbar puncture was not performed.

At that time, white blood cell count was 8.5G/L (Neutrophiles: 6.84G/L, Lymphocytes: 0.86G/L, Monocytes: 0.77G/L), C-reactive protein was 142 mg/L. Four blood cultures were performed, each were negative even with extended incubation time. Knee puncture yielded a purulent discharge, no germ nor crystal were spotted upon direct examination. Due to low prevalence of resistant *N.meningitidis* in France, the patient was first treated with amoxicillin 6 g/day.

Besides, HIV (human immunodeficiency virus) and HCV (hepatitis C virus) RT-PCR were negative. Syphilis and HBV (hepatitis B virus) serologies were negative. Chlamydia and Gonococcal RT-PCR in urine were negative. The urine culture remained negative. RT-PCR for SARS-CoV-2 was also negative, more than 2 weeks after the onset of first symptoms. At this stage, Henoch-Schönlein purpura was suspected.

The knee fluid culture was performed according to our hospital standardized protocols upon arrival at the laboratory. Direct examination was performed after Gram and May-Grünwald Giemsa stains but was negative. As for cultures, one run was performed in Schaedler’s anaerobe broth for 14 days, two others were performed on blood agar and chocolate agar in aerobic and aero-anaerobic conditions for 10 days. The aerobic culture was positive 2 days later and was identified as *Neisseria meningitidis*. A sample was then sent to the French National Reference Center at the Pasteur Institute in Paris for *N.meningitidis * for further testing in an appropriate transport media. Slide agglutination serogrouping led to identify Serogroup B. It was performed, as well as antimicrobial susceptibility testing, in accordance with international guidelines based on the French Microbiology Society and the Clinical and Laboratory Standards Institute guidelines, as described in chapter 6 and 11 of the 2011 World Health Organization “Laboratory Methods for the Diagnosis of Meningitis caused by *Neisseria meningitidis*” [11]. As such, a 4 mm depth Mueller-Hinton broth agar plate with 5% of sheep blood was used to perform a Kirby-Bauer disk diffusion test to evaluate the strain sensitivity to penicillins, cefotaxime, ciprofloxacin, chloramphenicol, rifampicin and azithromycin. Minimal inhibitory concentrations were determined using both disk diffusions and gradient strips in a 5% CO2 enhanced atmosphere. 

As the strain had intermediate sensitivity to amoxicillin and penicillin G, the patient was finally treated with cefotaxime 9 g/day for 2 weeks and surgical drainage. The drain was removed 2 days after due to absence of fluid and local improvement. Other cases of PMA were treated with similar protocols [3, 5]. Family and contacts were treated with prophylactic rifampicin for 2 days.

The patient improved 1 month after the treatment. The knee was painless, without any further impairment.

## Discussion and conclusions

Vaccination coverage for *N.meningitidis* serogroup C is low in France and consequently, our patient was not vaccinated. Besides data on the effect of vaccination on PMA is missing, most probably because only few cases are described. The most prevalent serogroup in France is the serogroup B [3], which can explain its presence in our case. This serogroup also fits the nosological frame of chronic meningococcemia associating cutaneous and rheumatologic symptoms mostly, with irregular fever and presence of *N.meningitidis * in fluids [4, 6]. No specific *N.meningitidis * serogroup seems to be associated with a higher probability of PMA [5]. Furthermore, septic arthritis is usually mutilating, for example *Staphylococcus aureus * or *N.meningitidis * itself in the nosological frame of invasive meningococcal disease, is associated with a poor outcome in a significant number of cases [1]. However, PMA seems to have a far better outcome than usual septic arthritis, even though serogroup C seems to be less aggressive and associated with an even better outcome [2]. This may suggest that PMA is, for some part, immune-mediated, with the presence of *N.meningitidis* being the trigger of such immune response, or even being incidental.

The early laboratory analysis showed no sign of immunodeficiency and there is no evidence of any immunocompromising condition in this young patient, in particular no spleen malfunction, nor any complement anomaly. Protein electrophoresis did not show any abnormality except for non-specific inflammation. The familial history of autoimmunity, especially the possible psoriasis in the mother, and a favorable undetermined genetic condition may be an explanation for this peculiar clinical situation, as hypothesised by Lavoipierre et al. in their own case report [5]. On the contrary, the results of Barahona et al. show that there is no higher prevalence in immunocompromised people, neither innate nor adaptive immunodeficiency, as most PMA cases occur in immunocompetent patients [2]. Even though acquired immunodeficiency is more and more prevalent with the ever growing use of immunomodulating treatments, and despite the rise of incidence of immunocompromising conditions such as diabetes, we do not observe any rise in the number of PMA. This may suggest that PMA are not linked to any specific immunodeficiency. On the contrary, we may conclude that PMA are the result of a rare combination of a propitious immune terrain and an acute condition allowing colonizing *N.meningitidis * to enter the bloodstream, thus strengthening the hypothesis of a dysimmunity-related condition rather than a symptom of actual immunodeficiency. 

 Saint-Quentin hospital takes care of a large territory as it stands in a mostly rural area. Farmers and hunters constitute a significant part of our patients. Bone and joint symptoms are quite common as traumas are frequent and prevalence of zoonotic diseases presenting with such symptoms is one of the highest in France. Diabetes also has a high frequency in our region, which has already been described as an underlying condition in another PMA case [12] but was not found in our patient. Furthermore, this case took place during the second COVID-19 wave in France, and our hospital was heavily hit and involved in helping our closest large hospitals by admitting a part of their ever growing number of inpatients. Thus, such a patient flow might have facilitated the occurrence of such a rare case.

Jovani et al. and Yokogawa et al. raised concerns about misdiagnosing other classic aetiologies of arthritis, such as crystal arthritis and psoriatic arthritis [8, 9]. Our patient did not have any risk factors for crystal arthritis and synovial fluid examination did not find any crystals. With regards to our patient’s familial history, a first bout of psoriatic arthritis may be hypothesized, but no skin or nail specific lesions were reported by the patient nor found upon examination and clinical improvement was achieved without any specific treatment for psoriasis.

In conclusion, the main interest of this case is the link between COVID-19 and the onset of arthritis 1 week later. As there is no history of trauma nor any exposure to a confirmed case of meningitis, we may assume a blood-borne infection from meningococcal carriage, even though *N.meningitidis * was not found in any of the four blood cultures. No immunocompromising condition was found. The pathophysiology of the bloodstream involvement remains unknown [1]. With nasopharyngeal carriage of *N.meningitidis * being frequent [1, 5], we may presume that the impairment of our patient’s respiratory tract by the SARS-CoV-2 infection may have facilitated bloodstream involvement and eventually arthritis. Such rare but serious complications of COVID-19 should be investigated, as they may appear later in the course of the disease. Further research is needed to highlight the link between COVID-19 and *Neisseria meningitidis * arthritis.

## Data Availability

Data sharing is not applicable to this article as no datasets were generated or analysed during the current study.

## Abbreviations

PMA: Primary meningococcal arthritis
RT-PCR: Retro-trancriptase, polymerase chain reaction
HIV: Human immunodeficiency virus
HBV: Hepatitis B virus
HCV: Hepatitis C virus
COVID-19: Coronavirus disease, 2019
SARS-CoV-2: Severe acute respiratory syndrome coronavirus 2
